# Comparison of Ti–35Nb–7Zr–5Ta and Ti–6Al–4V hydrofluoric acid/magnesium-doped surfaces obtained by anodizing

**DOI:** 10.1016/j.heliyon.2020.e04762

**Published:** 2020-08-28

**Authors:** Bárbara A. Reis, Laiza MG. Fais, Ana L.R. Ribeiro, Luis G. Vaz

**Affiliations:** a Department of Diagnosis and Surgery, São Paulo State University (Unesp), School of Dentistry, Araraquara, São Paulo, Brazil; b Department of Dental Materials and Prosthodontics, São Paulo State University (Unesp), School of Dentistry, Araraquara, Sao Paulo, Brazil

**Keywords:** Materials science, Nanotechnology, Metallurgical engineering, Dentistry, Alloys, Titanium, Nanotubes, Magnesium, Dental implants

## Abstract

**Objectives:**

Development of a new generation of stable β alloy, free of aluminum or vanadium and with better biological and mechanical compatibility and evaluate the surface properties of Ti–6Al–4V and Ti–35Nb–7Zr–5Ta after anodization in hydrofluoric acid, followed by deposition of different electrolyte concentrations of magnesium particles by micro arc-oxidation treatment.

**Methods:**

Disks were anodized in hydrofluoric acid. After this first anodization, the specimens received the deposition of magnesium using different concentration (8.5% and 12.5%) and times (30s and 60s). The surface morphology was assessed using scanning electron microscopy, and the chemical composition was assessed using energy dispersive x ray spectroscopy. The surface free energy was measured from the contact angle, and the mean roughness was measured using a digital profilometer.

**Results:**

Anodization in hydrofluoric acid provided the formation of nanotubes in both alloys, and the best concentration of magnesium considered was 8.5%, as it was the condition where the magnesium was incorporated without covering the morphology of the nanotubes. X-ray dispersive energy spectroscopy showed magnesium incorporation in all conditions. The average roughness was increased in the Ti–35Nb–7Zr–5Ta alloy.

**Conclusions:**

It was concluded that anodizing could be used to deposit magnesium on the surfaces of Ti–6Al–4V and Ti–35Nb–7Zr–5Ta nanotubes, with better results obtained in samples with magnesium concentration in 8.5% and the process favored the roughness in the Ti–35Nb–7Zr–5Ta group.

## 1 Introduction

Titanium is used as a biomaterial in the manufacture of dental implants due to its properties of low density, high mechanical strength, high corrosion resistance and excellent biocompatibility [1]. These properties can be improved by combining other elements with titanium, forming titanium alloys such as Ti–6Al–4V (TI-6AL-4V), which are well known for use in dental implants. Currently, there is considerable interest in the development of β titanium alloys, as they have favorable characteristics for dental applications, due to their combination of high strength and toughness [2].

However, concerns about possible toxicity of the Ti–6Al–4V alloy, and its mechanical incompatibility due to the difference in the elastic modulus relative to the cortical bone, have motivated the development of new alloys since 1985 [1]. Some studies associate the release of vanadium and aluminum ion to cytotoxic effects, peripheral neuropathy, osteomalacia and neurological disorders such as Alzheimer's [3]. Moreover, evaluation of the mechanical properties have demonstrated that the difference between the modulus of elasticity of pure titanium (~100–110 GPa), Ti–6Al–4V (~110 GPa) and cortical bone (~10–30 GPa) [4] may compromise the load transfer to the adjacent bone, causing eventual failure of the implant [5].

Thus, considerable efforts are being directed toward the substitution of alloying elements (Al and V) for others considered, at the present time, as non-toxic [6]. Among the studied alloys, those with added zirconium (Zr), niobium (Nb) and tantalum (Ta) have been considered the most promising [7]. Zr acts as a neutral element, forming a homogeneous solid solution in the α and β phases. Nb is a ductile and malleable metal that, in small amounts, improves the mechanical properties of the alloy significantly. Moreover, it is a β phase stabilizer element that may even decrease the modulus of elasticity [8], which makes a titanium alloy based on Nb suitable for use in implants. Ta also reduces the elastic modulus, when associated with commercially pure titanium, getting closer to the value of the bone modulus of elasticity (~60 GPa) [9, 10].

Surface properties are also of great importance in the performance of dental implants, especially with regard to the quality and accelerate the osseointegration. Osseointegration results in the stable and functional connection between the bone and a titanium surface. It strongly depends on the properties of the oxide film (passive film), which forms naturally on the surface of some metals [1]. This passive layer can be modified and improved by processes that affect ionic adsorption, absorption of proteins and cell-surface interaction, which are relevant to the functionality of the device. Presumptive that with the titanium oxide layer, surface properties such as chemical composition, surface energy, topography, roughness and wettability are changed [11]. Various surface modification processes are used, including grit-blasting, acid etching [12], plasma spraying and anodization [13].

Electrochemical surface modificationof titanium and its alloys is associated with occurring thin (2–5 nm) oxide layer of TiO2 which is formed spontaneously as a result of exposure of titanium and its alloys on air. The oxide layer with strong barrier properties protects titanium surface against corrosion [7]. Electrochemical surface modification of titanium is able to modify the layer of the titanium oxide film, making it more adherent and porous, with better biological properties [14]. The mechanism for modifying the oxide layer under anodizing conditions is well known [15]. Implant surfaces subjected to anodic oxidation, have morphological characteristics that facilitate adhesion, orientation and bone formation more quickly, allowing the insertion of implants in regions with low densities, accelerating the loading of implants [14].

A well-known process is surface modification, using electrochemical anodization based on hydrofluoric acid [11]. This process is capable of creating nanotubes on titanium surfaces, enhancing the bone growth, increasing bone cell responses *in vitro* and *in vivo* and inhibiting the responses of inflammatory cells when compared with non-anodized surfaces [11, 16]. In addiction, nanotubes in metals have been demonstrated as excellent candidates for the design of therapeutic implants, not only because nanotubes structures support tissue integration, but also because nanotubes act as remarkable reservoirs for slow drug elution over extended time periods [16].

A strategy for improving titanium surface involves the biofunctionalization of implant surfaces with elements that participate in the osseointegration process and are present in bone, such as magnesium [17]. Magnesium has been shown to positively influence the differentiation of progenitor cells into osteoblast cells as well as to enhance the environmental and osteogenic bone anchor [17, 18]. However, there is still no well-established protocol for the best management of this doping, requiring further studies with different concentration of the electrolyte to establish appropriate levels [18].

The coating of the biomaterial by the porosities, similar to bone matrix, followed by the addition of magnesium, which is a native element of the human body, significantly influences the strength of the union between bone tissue and the implant. The similarity of the composition and conformation of the bone mineral component, results in a strong chemical and mechanical establishes between the implant and the surrounding tissue, promoting a more adequate bone fixation [19], which favors the use of biomaterial in critical receiving areas, such as regions with poor bone availability or poor bone (type IV).

Thus, since both the chemical composition and topography of the surface are crucial for apposition and bone formation around the implant [1, 13], the objective of this study was to promote surface treatments in the Ti–6Al–4V and Ti–35Nb–7Zr–5Ta alloys, to obtain a homogeneous film with nanotube morphology and chemical addition of magnesium particles, followed by the evaluation of the morphological characteristics, chemical composition, wettability and roughness, so that in the future, from obtaining a stable film with this work, we can continue research in the biological area.

## 2 Materials and methods

TI-6AL-4V Two titanium alloys were used in this study, Ti–6Al–4V (TI-6AL-4V) and Ti–35Nb–7Zr–5Ta (%wt) (TNZT). TI-6AL-4V discs (8mm Ø x 2mm thickness) were obtained by machining commercial bars (Realum Indústria e Comércio de Metais Puros e Ligas Ltda, São Paulo, Brazil). The starting materials to obtain TNZT alloy (Ti, Nb, Zr, and Ta, with a degree of purity greater than or equal to 99.00%) were arc melted in an argon atmosphere, remelted (3–5 times) to ensure homogeneity and, vacuum heat-treated (1000 °C, 8 h, furnace cooled). They were hot-swaged into bars (≈11 mm Ø) and machined into discs with 8 mm diameter and 2 mm thick. These discs were vacuum heat-treated at 1000 °C for 1 h and air-cooled to relieve stress and tensions [20].

Disks were mechanically polished with silicon carbide abrasive paper (T 216, Norton Abrasives Brazil) with #120, #320, and #600, #1200 and #2000 (for 40 s each). They were cleaned in an ultrasonic bath with isopropyl alcohol for 10 min and air-dried and finally they were etched for 8 s in Kroll's solution (distilled water, 75% nitric acid, and 45% hydrofluoric acid; 1:1:1 in vol) to remove the passive oxide layer [10].

The disks were divided into two groups TI-6AL-4V (Ti–6Al–4V) or TNZT (Ti–35Nb–7Zr), and assigned to subgroups as listed in Table 1. TI-6AL-4V.

**Table 1:** Experimental subgroups, electrolytes and anodizing parameters.

Subgroups	Electrolytes	Anodizing parameters
Control-	-	-
Control+	0,3 mol/L hydrofluoric acid(12) 0% magnesium acetate	60 min, 20 V, 2 A
HFMg760	0,3 mol/L hydrofluoric acid and 8.5% magnesium acetate(18)	60 min, 20 V, 2 A 1 min, 200 V, 2 A
HFMg730	0,3 mol/L hydrofluoric acid and 8.5% magnesium acetate	60 min, 20 V, 2 A 30 s, 200 V, 2 A
HFMg160	0,3 mol/L hydrofluoric acid and 12.5% magnesium acetate(18)	60 min, 20 V, 2 A 1 min, 200 V, 2 A
HFMg130	0,3 mol/L hydrofluoric acid and 12.5% magnesium acetate	60 min, 20 V, 2 A 30 s, 200 V, 2 A

Anodization was carried out with magnetic stirring, using the potentiostatic method and 140 mL of each electrolyte at room temperature of 25 °C. The disks (anode) remained at 8 mm distance from the cathode (stainless steel plate). After each anodization, the disks were cleaned in an ultrasonic bath with isopropyl alcohol for 10 min and air-dried.

The surfaces was characterized by scanning electron microscopy (SEM, JEOL JSM-6610LV, Tokyo, Japan) with secondary electrons and high-resolution field emission microscopy (FEG-JSM-7500F, JEOL, Tokyo, Japan).

The chemical composition of the surfaces was assessed by an Energy Dispersive X-ray Spectroscopy (EDS) coupled to the FEG. Discs were placed directly onto the stub and examined without any preparation or manipulation.

The surface free energy (SFE) was analyzed using the sessile drop method with a goniometer (Ramé-Hart 10000; Ramé-hart instrument co.) with different treatments using the SCA-20 software with the Young-Laplace equation [21]. The contact anglewas measured using fluids differing in hydrophobicity (distilled water, glycerol, and diiodomethane) [21] at a controlled temperature (25 °C) and after the settling time (30s) of the drop (15 μL). The disks of each subgroup (N = 100) were measured 3 times, and the average of each surface and fluid was analyzed as described by Owens and Wendt [22] in the software SCA 20 (DataPhysics Instruments GmbH).

The surface roughness was measured with a roughness analyzer (Surftest SJ-401; Mitutoyo Corp) with an accuracy of 0.01 μm, a read length of 2.4 mm and a speed of 0.5 mm/s . Five measurements of Ra (mean roughness) were performed on each surface, with nine discs for each subgroup, and the mean values were calculated.

Data were analyzed using the software GraphPad Prism 7. The data normality distribution was tested by Shapiro-Wilk. Two-way ANOVA with Tukey post-test was used to clarify whether titanium alloys (TI-6AL-4V and TNZT) or surface modifications.The level of significance was set at 5%.

## 3 Results

**Figure 1:**
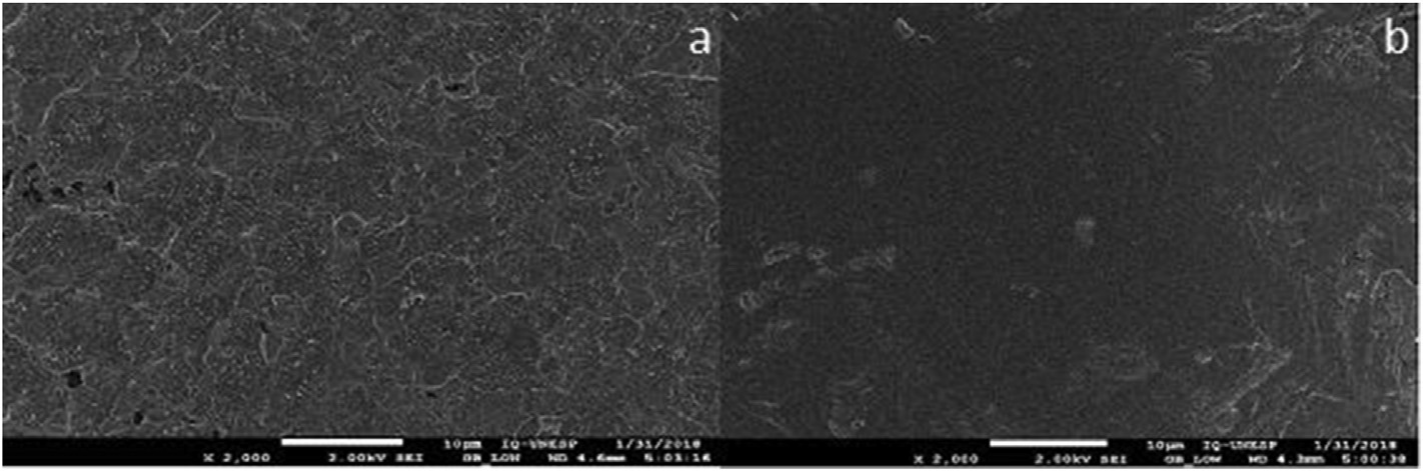
Scanning electron microscopy images of controls - groups: a – TI-6AL-4V without treatment; b – TNZT without treatment. TI-6AL-4V.

The initial etching in Kroll solution resulted in surfaces with simplified topography showing only granules (Figure 1a and Figure 1b).

**Figure 2:**
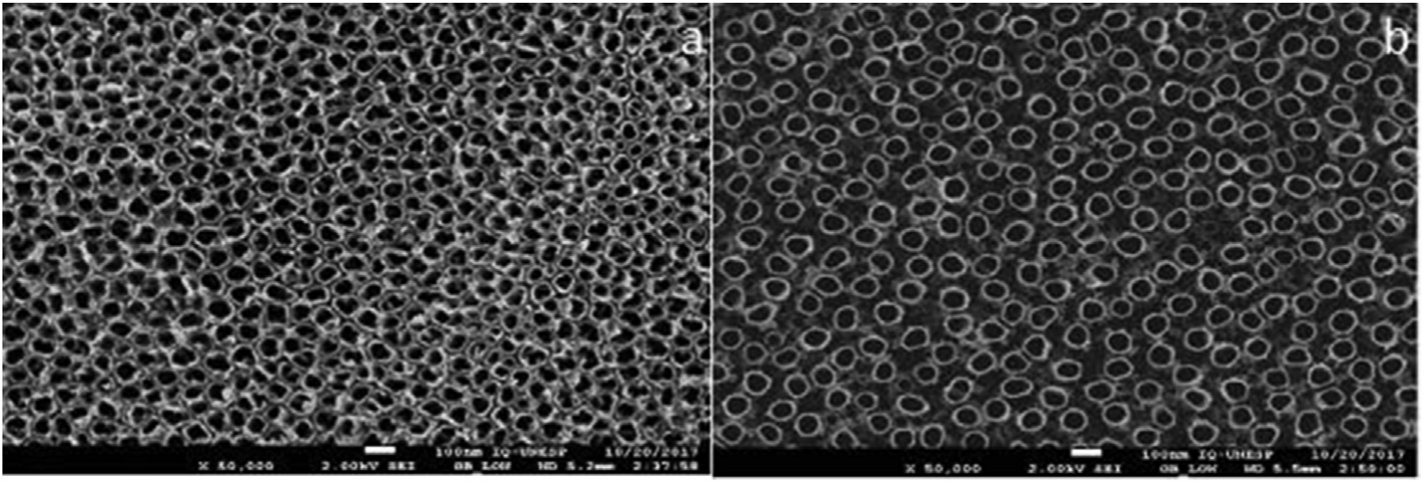
Scanning electron microscopy images of controls + groups a – TI-6AL-4V HF; b – TNZT HFTI-6AL-4V.

**Figure 3:**
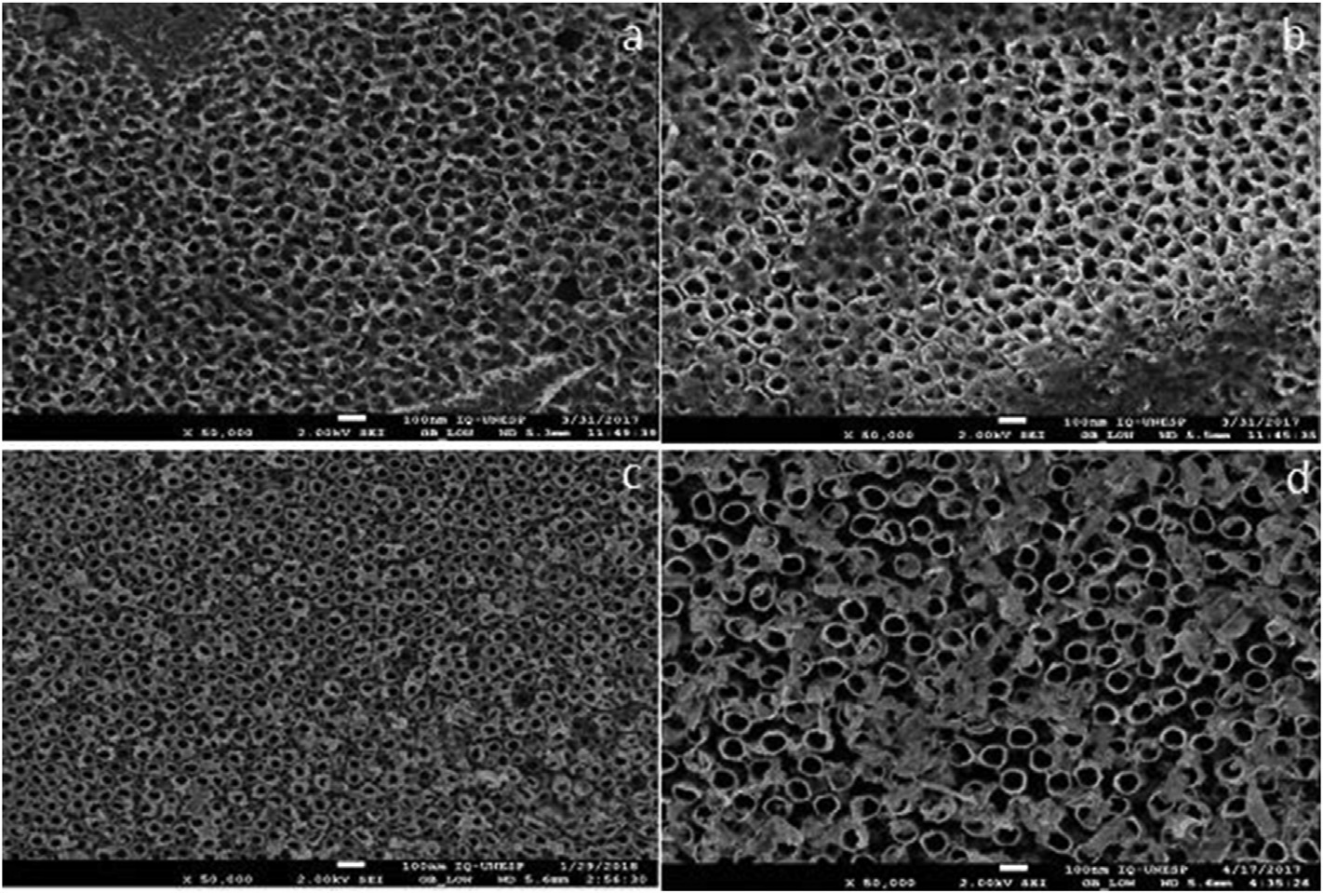
Scanning electron microscopy images of groups anodized with 0.07 mol/L Mg2+: a – TI-6AL-4V HFM730; b – TI-6AL-4V HFM760; c – TNZT HFM730; d – TNZT HFM760.

**Figure 4:**
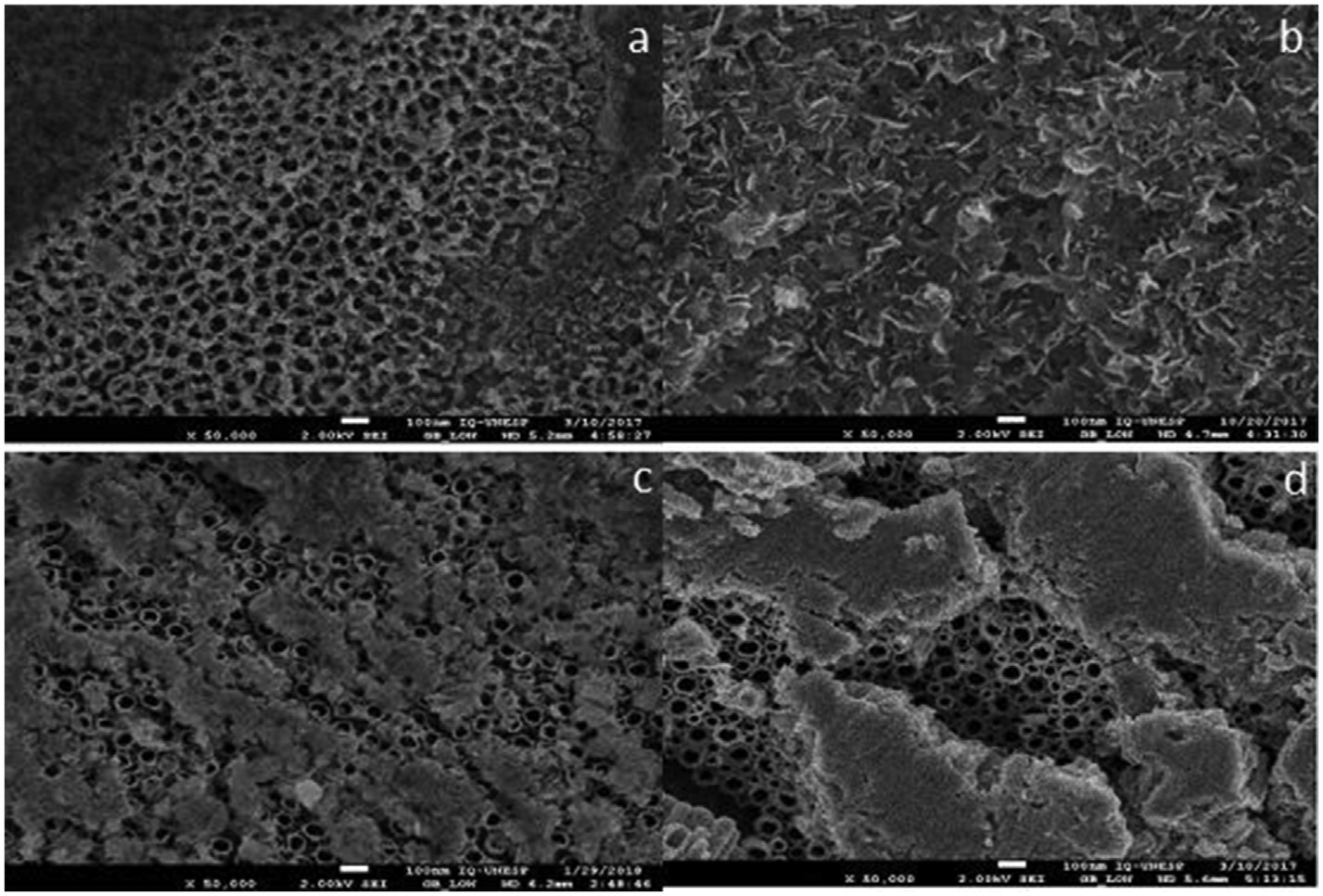
Scanning electron microscopy images of groups anodized with 0,1 mol/L Mg2+: a – TI-6AL-4V HFM730; b – TI-6AL-4V HFM760; c – TNZT HFM730; d – TNZT HFM760.

Electrochemical surface modification with hydrofluridic acid resulted in the formation of nanotubes (Figures 2a and 2b). The second anodization with Mg resulted in a different structure in both alloys depending on the electrolyte concentration. In all subgroups of anodized specimens with 8.5% Mg concentration it was possible to identify the nanotubes in the first layer (Figure 3). In the subgroups with 12.5% Mg electrolyte concentration few or no nanotubes were identified (Figure 4).

The EDS technique identified only the alloy components in the control^−^ and control^+^ subgroups. Magnesium was incorporated in all specimens anodized with magnesium acetate, and at 12.5% concentrations, the equipment detected fewer alloy elements, possibly because the magnesium layer was thicker (Table 2).

**Table 2:** Results of EDS analysis of specimens anodized with different concentrations of electrolyte for TI-6AL-4V and TNZT alloys.

Element	Liga TI-6AL-4V		Liga TNZT	
	Anodized 8,5%(at.%)	Anodized 12.5%(at.%)	Anodized 8,5%(at.%)	Anodized 12.5%(at.%)
Ti	33.04	29.14	24.31	19.24
Al	3.85	2.88	-	-
V	1.45	0.89	-	-
Mg	11.81	17.49	7.11	12.36
O	48.85	48.60	57.6	58.96
Ta	-	-	0.65	0.51
Nb	-	-	7.81	6.46
Zr	-	-	1.52	1.47

There were no significant differences between the alloys in relation to SFE values (Table 3), but betwewn the groups that had the highest SFE were TNZT HF M 760 and TNZT HF M130. There was a significant difference in Ra between the alloys, and the TNZT alloy showed higher Ra values. Among the treatments, the TNZT HF M730 group had the highest Ra value within the TNZT group, while the control^−^ had a value of 0.27 (see Table 4).

**Table 3:** Means and standard deviation of surface free energy (SFE, in mN/m) determined using theOwen-Wendt-Rabel-Kaelble method.

	TI-6AL-4V	TNZT
Control^-^	43.6 ± 1.9 ^Ab^	47.7 ± 0.7 ^Bb^
Control^+^	50.7 ± 0.7 ^Aa^	54.0 ± 1.2 ^Bab^
HF M 730	51.6 ± 3.7 ^Aa^	50.5 ± 3.5 ^Bab^
HF M 760	54.4 ± 2.0 ^Aa^	56.9 ± 1.4 ^Ba^
HF M130	49.7 ± 2.5 ^Aab^	54.6 ± 3.1 ^Ba^
HF M 160	49.4 ± 2.4 ^Aab^	51.0 ± 4.3 ^Bab^

Upper case superscript letters indicate significant differences between the lines (P < .05).

Lower case superscript letters indicate significant differences between the columns (P < .05).

**Table 4:** Means and standard deviation of the mean roughness (Ra, in nm) according to the control and experimental subgroups in HF.

	TI-6AL-4V	TNZT
Control^-^	0.16 ± 0.03 ^Ba^	0.27 ± 0.03 ^Ad^
Control^+^	0.14 ± 0.00 ^Ba^	0.35 ± 0.06 ^Ac^
HF M 730	0.13 ± 0.01 ^Ba^	0.51 ± 0.01 ^Aa^
HF M 760	0.12 ± 0.02 ^Ba^	0.43 ± 0.03 ^Ab^
HF M130	0.13 ± 0.01 ^Ba^	0.43 ± 0.02 ^Ab^
HF M 160	0.12 ± 0.01 ^Ba^	0.40 ± 0.03 ^Abc^

Upper case superscript letters indicate significant differences between the lines (P < .05).

Lower case superscript letters indicate significant differences between the columns (P < .05).

## 4 Discussion

**Figure 5:**
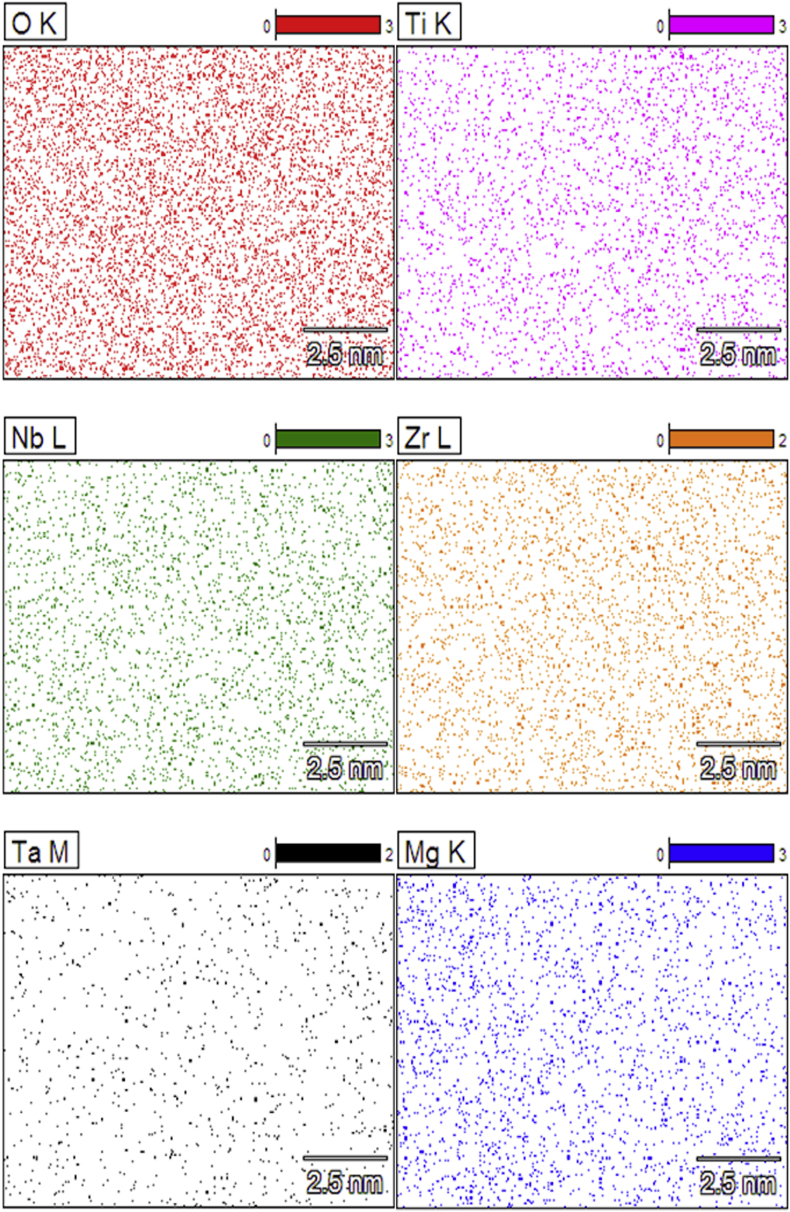
High-resolution EDS mapping in specimen TNZT HFM730. Presence of O, Ti, Nb, Zr, Ta and Mg.

It is known that when Ti is in contact with oxygen, a layer of oxide forms on its surface, approximately ≤10nm thick, which gives the metal protection and Ti alloys may have varied oxide composition, depending on the alloy elements present which may modify the protective property of Ti oxide [7]. The alloy elements of this study are known for many advantages, such the generate Nb2O5 by the presence of Nb, Zr, ZrO2, Ta, Ta2O5, and in the alloy TI-6AL-4V, V create VO2 and Al, Al2O3. The oxides of Ti modified by the oxides of Nb, Zr and Ta have better stability and therefore may provide better protection than Al and V oxides [7, 23]. Which presents results of EDS analysis (Table 2), the part of Nb, Zr and Ta is coming from the substrate due beam penetration, one can note that amounts of that elements are detection in limit of the EDS equipment, and in addition, the mapping shows the coincidence of the alloy elements with the oxygen (Figure 5), being possible the presence of Nb2O5, ZrO2 and Ta2O5.

The nanotubular structure of the TiO2 provides not only a nanotopographical surface to foster bone formation, but also space for delivery of targeting agents to attain extra functions, including antibacterial agents, bone growth enhancement agents, and anti-inflammatory agents have been incorporated into nanotubes in this respect [24]. Magnesium was incorporated into the surfaces with the intention of acting as bone growth agent, for the reason that it is relevant for bone formation because enhancement of the early osteoblastic cell response is essential for subsequent favorable osteoblast behaviors and ultimately the in vivo bone healing of Ti implants [25], however, the electrochemical anodizing process with magnesium does not promote a self-organized morphology in titanium [18].

An organized structure of nanotubes is obtained by anodizing with 0.3 mol/L in hydrofluoric acid resulted in the formation of, which structure acts in osseointegration [12, 26]. Oh et al. [12] showed that the structure of titanium nanotubes promotes a 400% presumptive in cell adhesion to the implant, by mechanisms such as vertically aligned TiO2 nanotubes exhibit enormously larger surface areas than the flat Ti surface and the pronounced vertical topology contributes to the interlocked cell configuration. Ellingsen et al. [11] suggested that fluoride can be displaced by the oxygen bound to the phosphate [PO4^3-^] of the physiological fluid through the ion exchange. This generates the covalent bond between the titanium implant and the newly formed peri implant tissue, generating greater adhesion between the implant and the receptor tissue.

Following the formation of nanotubes, magnesium was incorporated into the oxide layer. Magnesium is known to stimulate the production of calcitonin, which helps to preserve bone structure and suppress the action of parathyroid hormone. This reduces bone resorption, in addition to stimulating the enzymatic reaction required for the formation of the new bone which are magnesium dependent [17]. Oliveira et al. [18] obtained good results with the incorporation of Mg over calcium/phosphorus surfaces varied electrolyte concentration from 0 to 12.5% using magnesium acetate tetrahydrate (Sigma-Aldrich Co; St Louis, Mo, USA). In electrochemical surface modification, both intrinsic factors (electrolyte compositions) and extrinsic factors (electrical parameters, and electrolyte temperature) affect the formation and microstructure of electrochemical surface modification [13], so in the present study 3 electrolyte concentrations were tested, being 0%, 8.5% and 12.5%. Therefore, in order to maintain the favorable characteristics of the magnesium deposition without significant interference in the pattern of nanotubes previously formed by the HF, different condition for incorporating Mg were used.

Oliveira et al. [18] modified titanium surfaces with calcium, promoting pores, and subsequently succeeded in incorporating 12.5% magnesium without cover the initial morphological change. The morphological analysis of this study, performed using high resolution SEM, showed that the magnesium was incorporated and maintained the initial morphology in the specimens with 8.5% concentration of Mg in the electrolyte (Figure 3), unlike the condition found in the subgroups having 12.5% concentration that covered almost all nanotubes (Figure 4). In the EDS result (Table 2), magnesium detection was higher in the 12.5% group in TI-6AL-4V and TNZT, detecting fewer alloy elements, which justifies this large coverage over the nanotubes, which makes it difficult to detect alloy elements. This shows that the electrolyte concentration is relevant, and should be studied and researched to achieve a standard for anodizing.

Intrinsic factors of electrolytes, as composition of the solution, may be influence the medium of current conduct. Higher Mg electrolyte concentration cause the current to remain longer at its maximum value, probably due to the increase in conductivity of the electrolyte [13]. Theoretically, this would influence the total amount of oxide formed during the process, which would affect the thickness and/or the compaction of the oxide layers [18].

The electrolyte condition distinctly influenced the wettability of the surface, as the result was published by Huan et al. [26] when the contact angle values of NiTi alloy decreased to 70° for 15° after anodizing with hydrofluoric acid because the surface becomes more hydrophilic with the nanotubular morphology. SFE has an inverse tendency in relation to the contact angle: that is, the smaller the contact angle values, the higher the SFE. In this study, or both alloys, the treatments had a low difference in the SFE values, but with the results obtained the HFM760 group had higher values in both groups, and the control^−^ subgroup had the lowest values, so the use of Mg suggests that the condition in this subgroup has a positive influence on the characteristics designed previously using HF.

It is known that, in treatment with acids, the roughness of the implant becomes homogeneous due to the formation of the nanotubes; and, there is an increase of the surface area that, consequently, improves the possibility of bio adhesion of the cells [24]. Ellingsen et al. [11] verified the influence of fluoride to modify the surface of titanium in rabbit tibias. He did so by conducting quantitative analyses of surface roughness, biomechanical locking and in vivo tissue reaction. The implants modified using fluoride showed surfaces with less roughness than the control implants, a result similar to that found for the TI-6AL-4V alloy. However, the removal torque and the proportionality limits of Ellingsen et al. [11] between the bone and the implant, were significantly higher for the test group compared to the control group implants. The surface modification of implants, using fluoride, may result in significant morphological and physical-chemical phenomena for bone response. A possible explanation for these results is that changes in morphological structure and surface wettability can promote bone interaction, depending not only on the roughness value [11]. However, the roughness of the TNZT increased considerably with the anodizing carried out, especially in the HFM730 group. A positive result to be accepted is that rough and nanotubes surfaces have a more pronounced and beneficial influence on cellular activity.

Despite the limitations of the study, the lower concentration of Mg electrolyte maintained the proposed deposition of Mg with the maintenance of nanotube morphology, without negatively influencing SFE and roughness parameters. With more research, we believe the development of this biomaterial in the future will be interesting for use as short implants, which are not yet used with the safety of regular implants in implant dentistry, especially in areas of low density bone, because of the similarity of the composition and conformation of the bone mineral component, results in a strong chemical and mechanical establishes between the implant and the surrounding tissue, promoting a more adequate bone fixation [19], which favors the use of biomaterial in critical receiving areas, such as regions with poor bone availability or poor bone (type IV). Short implants are used more cautiously in the maxilla compared to the mandible, in situations of limited bone height (less than 8 mm), limited width (less than 7 mm) and poor quality (type IV bone). Short implants less than 8 mm in length have lower survival rates than standard implants [27]. More studies need to be done, mainly biological to verify the real influence of this physical-chemical modification on bone cells.

Based on the results of the present study, the TNZT alloy performs equal to or better than the TI-6AL-4V alloy, under the same condition of anodization. Moreover, as the osseointegration mechanism reinforced by magnesium surface chemistry is not fully understood at present, but we know that different concentrations of electrolyte can influence the morphology of the electrochemical surface modification and result in different effects on Ti alloy roughness and SFE. Although it has been shown that increased electrolyte concentration has a negative influence on structural homogeneous maintenance of nanotubes. Therefore, titanium alloys treated by electrochemical surface modification in 8.5% magnesium electrolyte had better results and need attention in more research.

## Declarations

### Author contribution statement

Bárbara A. Reis: Conceived and designed the experiments; Performed the experiments; Wrote the paper.

Laiza M. G. Fais: Performed the experiments; Analyzed and interpreted the data; Wrote the paper.

Ana L. R. Ribeiro: Performed the experiments.

Luis G. Vaz: Conceived and designed the experiments; Performed the experiments; Analyzed and interpreted the data.

### Funding statement

This research did not receive any specific grant from funding agencies in the public, commercial, or not-for-profit sectors.

### Competing interest statement

The authors declare no conflict of interest.

### Additional information

No additional information is available for this paper.
